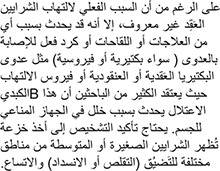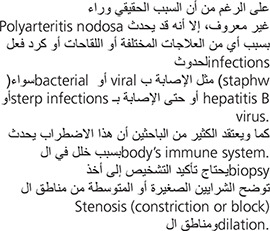# Embracing bilingualism in medical education: Integrating Arabic for enhanced understanding in the Arab world

**DOI:** 10.5339/qmj.2024.5

**Published:** 2024-02-13

**Authors:** Omar Ahmed Abdelwahab

**Affiliations:** Faculty of Medicine Al-Azhar University, Cairo, Egypt; Medical Research Group of Egypt, Negida Academy, Arlington, MA, USA Email: omar3240109@gmail.com

**Keywords:** Arabic, integrated education, medical education, bilingual teaching, Arabization

Optimal healthcare cannot be achieved without high-quality medical education. Language is the primary tool of education, containing the four learning cornerstones: reading, listening, speaking, and writing.^1^ The importance of using one’s mother tongue language in education, particularly in specialized fields like medicine, cannot be overstated. When students learn in their native language, they achieve a deeper and more intuitive understanding of complex concepts, as they are not hindered by the additional cognitive load of processing information in a foreign language.^2^ This methodology improves cognitive development and promotes a more profound emotional bond with the subject matter, resulting in enhanced retention and application of knowledge.^3^ Moreover, acquiring knowledge in one’s native language upholds and fosters cultural identity, establishing a comprehensive educational setting where students can succeed academically while preserving a profound link to their cultural heritage.^1,2^

Using the mother language in medical education is adopted in most countries globally, especially developed countries and even nations with less widely spoken languages. Regions such as Scandinavia, the Baltics, and others, including Iceland, Latvia, Estonia, and Lithuania, place significant importance on teaching medical education in their native languages.^4^ However, there is a notable disregard for the native language in medical education in African and Middle Eastern countries and the Arab world. Several factors pushed those countries against using the Arabic language in medical education, including historical antecedents and the practice of colonization.^4-6^ Additionally, many medical schools and university institutions are primarily opened and established by foreign-speaking countries, such as Lebanon in the Middle East region.^7^

However, Arabic is more than adequate to replace foreign languages in medical education because of the richness of medical terms and even roots from which medical terms can be derived.^8^

In most Arab countries, there is a gap between the educational and textbook languages. The textbook’s language is exclusively native English or another foreign language without any Arabic words. However, the practical academic language in teaching medicine in most Arab countries is a mix of Arabic and English or another foreign language (bilingual teaching), where most medical terms are spoken using the foreign language. However, all conversations and explanations are in Arabic, except for medical terms. However, some Arab countries deviate from the rule, such as Lebanon and Algeria, where the textbooks, conversations, and explanations are in the foreign language. Nevertheless, the medical terms are not a big bulk and cannot be the reason for not involving Arabic exclusively in the medical textbooks. Al-Sibai et al. conducted a study revealing that medical terminology makes up a mere 3.3% of medical literature, whereas the majority comprises general words.^5^

This is a trial of the first step involving the Arabic language in medical textbooks without any Arabization in the medical terms. This case study will involve two rare diseases in three linguistic formats: the full English text, the full Arabic text, and the suggested text (mixed text like traditional medical education in Arab countries). The choice of rare diseases (Alpha-1 antitrypsin deficiency and Polyarteritis nodosa) was made because it includes more specialized medical terms than the general medical terms, so if this approach succeeded with rare diseases, the involvement in other conditions would be easier. The approach is presented in Table 1.

We suggested this approach as an introduction for the first step in the complete Arabization of medical education, as the prevailing sentiments regarding Arabic as an instructional language in medical education are still unfavorable to both students and teachers.^8^ This is primarily due to the lack of proficiency in medical translators. However, medical students believed that including Arabic terminology in an exclusively English-based curriculum facilitated their learning.^9^ So, our approach solves this problem by keeping the medical terms without Arabization in this step. This approach was evaluated by Alsuliman et al. by comparing the achievement of medical students in multiple-choice questions for a provided paragraph if presented with the full English text, the full Arabic text, and the bilingual text.^10^ They found that a markedly greater average score and number of accurate responses were achieved in the shortest duration for the bilingual paragraph.

In conclusion, incorporating the Arabic language into medical education and medical textbooks in the Arab world serves to preserve cultural identity and strategically improve medical students’ educational journey and understanding. Our bilingual approach, which combines Arabic and English, recognizes the worldwide scope of medical science while acknowledging the significant influence of education in one’s mother tongue. This approach will help both Arab countries that use bilingual medical teaching and those that teach medicine using foreign languages. Our approach is not the suggested Arabization of the medical field and not the first step, but just an introduction to the first step in the process of Arabization.

## Conflict of Interest

None.

## Acknowledgment

I want to thank my colleague Abdullah Ashraf Hamad for starting the era of writing about Arabic involvement in medical education in the community of young medical researchers. I also thank Dr. Eman Gaber for helping in preparing the translated texts.

## Figures and Tables

**Table 1.** The suggested approach.

**The English text**	**The Arabic text**	**The suggested text**
Alpha-1 antitrypsin deficiency (A1AD) is a hereditary disorder characterized by low levels of a protein called alpha-1 antitrypsin (A1AT), which is found in the blood. This deficiency may predispose an individual to several illnesses and most commonly manifests as chronic obstructive pulmonary disease (including bronchiectasis) and liver disease (especially cirrhosis and hepatoma) or, more rarely, as a skin condition called panniculitis. A1AD is also more frequent among individuals with Wegener’s granulomatosis, now called polyangiitis with granulomatosis.	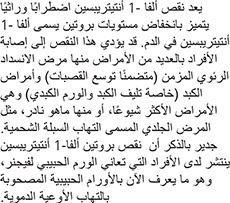	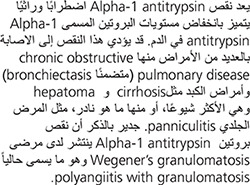
A deficiency of A1AT allows substances that break down proteins (so-called proteolytic enzymes) to attack various body tissues. The attack results in destructive changes in the lungs (emphysema) and may also affect the liver and skin.	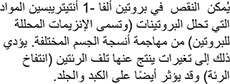	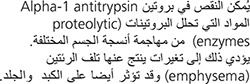
Alpha-1 antitrypsin is ordinarily released by specialized granules within a type of white blood cells (called neutrophils or polymorphonuclear leukocytes) in response to infection or inflammation. Deficiency of alpha-1 antitrypsin results in unbalanced (i.e., relatively unopposed) rapid breakdown of proteins (protease activity), especially in the supporting elastic structures of the lungs. Over the years, this destruction can lead to progressive emphysema and is accelerated by smoking, some occupational exposures, and likely by other genetic modifiers of this risk, which remain incompletely understood.	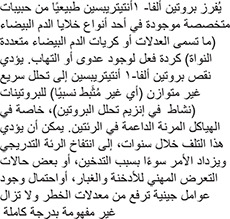	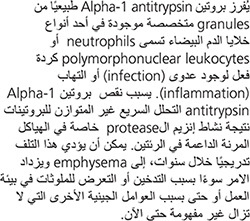
Polyarteritis nodosa is a rare multi-system disorder characterized by widespread inflammation, weakening, and damage to small and medium-sized arteries. Blood vessels in any organ or organ system may be affected, including those supplying the kidneys, heart, intestine, nervous system, and/or skeletal muscles. Damage to affected arteries may result in abnormally increased blood pressure (hypertension), “ballooning” (aneurysm) of an arterial wall, the formation of blood clots (thrombosis), obstruction of blood supply to certain tissues, and/or tissue damage and loss (necrosis) in certain affected areas.	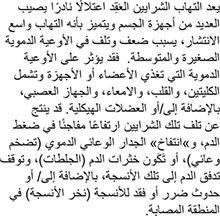	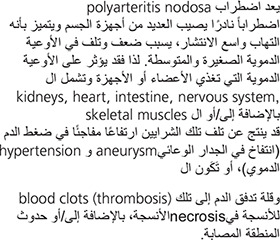
The disorder is more common among men and is more likely to present during early middle age, between 40 and 50 years.	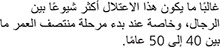	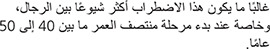
Although the exact cause of polyarteritis nodosa is unknown, it is clear that an attack may be triggered by any of several drugs or vaccines or by a reaction to infections (either bacterial or viral) such as strep or staph infections, or hepatitis B virus. Many researchers suspect the disorder is due to disturbances in the body’s immune system. Confirming the diagnosis required either a biopsy showing small or medium-sized arteries with alternating areas of stenosis (constriction or block) and dilation.